# S28 peptidases: lessons from a seemingly 'dysfunctional' family of two

**DOI:** 10.1186/1741-7007-8-87

**Published:** 2010-06-28

**Authors:** John W Kozarich

**Affiliations:** 1 ActivX Biosciences, Inc., 11025 North Torrey Pines Road, La Jolla, CA 92027, USA

## Abstract

**Abstract:**

A recent paper in *BMC Structural Biology *reports the crystal structure of human prolylcarboxypeptidase (PRCP), one of the two members of the S28 peptidase family. Comparison of the substrate-binding site of PRCP with that of its family partner, dipeptidyl dipeptidase 7 (DPP7), helps to explain the different enzymatic activities of these structurally similar proteins, and also reveals a novel apparent charge-relay system in PRCP involving the active-site catalytic histidine.

See research article: http://www.biomedcentral.com/1472-6807/10/16/

**Commentary:**

The S28 serine peptidase family is something of an enzymatic odd couple. While showing low sequence similarity to all proteins except each other, the two known family members appear to be at odds functionally; one, prolylcarboxypeptidase (PRCP), is a carboxypeptidase that cleaves single hydrophobic residues from the carboxyl termini of proteins that end with a Pro-X motif (where X is any hydrophobic amino acid), while the other, human dipeptidyl peptidase (DPP7), is an aminopeptidase that cleaves amino-terminal X-Pro dipeptides. The structural basis of this orthogonal specificity would undoubtedly be interesting, and a recent report in *BMC Structural Biology *from the Merck Global Structural Biology group (Soisson *et al*. [1]) has now met that expectation. In addition they reveal a new wrinkle to the iconic catalytic triad common to most serine hydrolases.

The practical pharmaceutical interest in both these enzymes as potential drug targets is at present speculative. PRCP can inactivate a number of peptide hormones, such as angiotensin II, III and prekallikrein, implicating a role for the enzyme in hypertension, tissue proliferation and smooth-muscle growth. These properties suggest that this enzyme may well be a useful target for hypertension and anti-inflammatory therapy [2]. Another (non-S28 family) dipeptidyl dipeptidase (DPP4) is a major drug target in type 2 diabetes, and Merck has already developed a successful inhibitor of DPP4, the anti-hyperglycemic drug sitagliptin, for the treatment of type 2 diabetes. The DPP enzymes are rich in biological functions and other drug targets emerging from the group are possible [3].

## A peptidase with a difference

Soisson *et al*. [1] have solved the crystal structure of human PRCP. Their refined model reveals two major domains - a prototypical α/β hydrolase fold derived from two non-contiguous stretches of the protein and a novel helical bundle (referred to as an SKS domain by the authors) that serves to cap the active site. The α/β hydrolase fold is a tertiary fold adopted by many proteins that have no obvious sequence similarity but ultimately diverged from a common ancestor [4]. In addition, a dimerization interface is observed that is consistent with the biochemical properties of PRCP in solution. The arrangement of the catalytic triad of amino acids in the active site (Ser179, Asp430 and His455) is a classic constellation seen in other serine α/β hydrolases [4]. The spatial arrangement of these three amino acids in the active site enables them to form hydrogen bonds with each other and with the substrate and cooperate in covalent catalysis by the serine.

**Figure 1:**
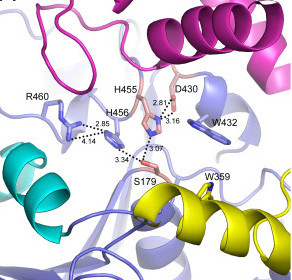
**A new charge-relay motif for serine proteases**. Part of the structure of the active site of PRCP as determined by Soisson *et al*. [1] is shown here. The classic catalytic triad comprises Ser179 (S179), His455 (H455) and Asp430 (D430), which are linked by hydrogen bonds (H bonds, dotted lines). In the PRCP active site, the catalytic H455 is also linked via S179 to H456, which is in turn H-bonded to Arg460 (R460). Thus, these five residues form a potential charge-relay system. A similar structure is present in DPP7 (PDB 3JYH).

The active site does have one surprise - an apparent charge-relay system that links the catalytic histidine (His455) with His456 and Arg460 and might even suggest a kind of dual catalytic triad bifurcated off Ser179, the ultimate nucleophile (Figure 1). The function of this unique system is unknown. The authors suggest that the charge perturbation of the His456/Arg460 diad may explain the acidic pH optimum of PRCP; that sounds reasonable enough, but some deeper involvement in the catalytic mechanism is a tantalizing prospect to ponder. A similar adjacent His-His motif, lacking the Arg relay, is present in serine lipases [5]. Future work on the function of this unique catalytic array will be worth following.

The substrate specificity for a penultimate proline at the carboxyl terminus is explained by the presence of a hydrophobic S1 binding pocket, formed by two Met and two Trp residues, adjacent to the catalytic serine. This appears to be a variation of similar Pro-specific S1 sites that are found in other prolylpeptidases, such as the pharmaceutically important DPP4 [6].

## Family comparisons

The structural comparison by Soisson *et al*. [1] of PRCP to its family partner, the DPP7 aminopeptidase, was made possible by the deposition by the Structural Genomics Consortium of the coordinates for the structure of DPP7 in October 2009 (PDB 3JYH); these findings have not yet been published. Essentially all of the structural features observed for PRCP and described above are preserved between the two enzymes. With a backbone root mean square deviation (r.m.s.d.) for the aligned structures of 1.20 Å, the structural comparison of PRCP and DPP7 described by Soisson *et al*. thus serves to define a unique fold architecture for the S28 family.

**Figure 2:**
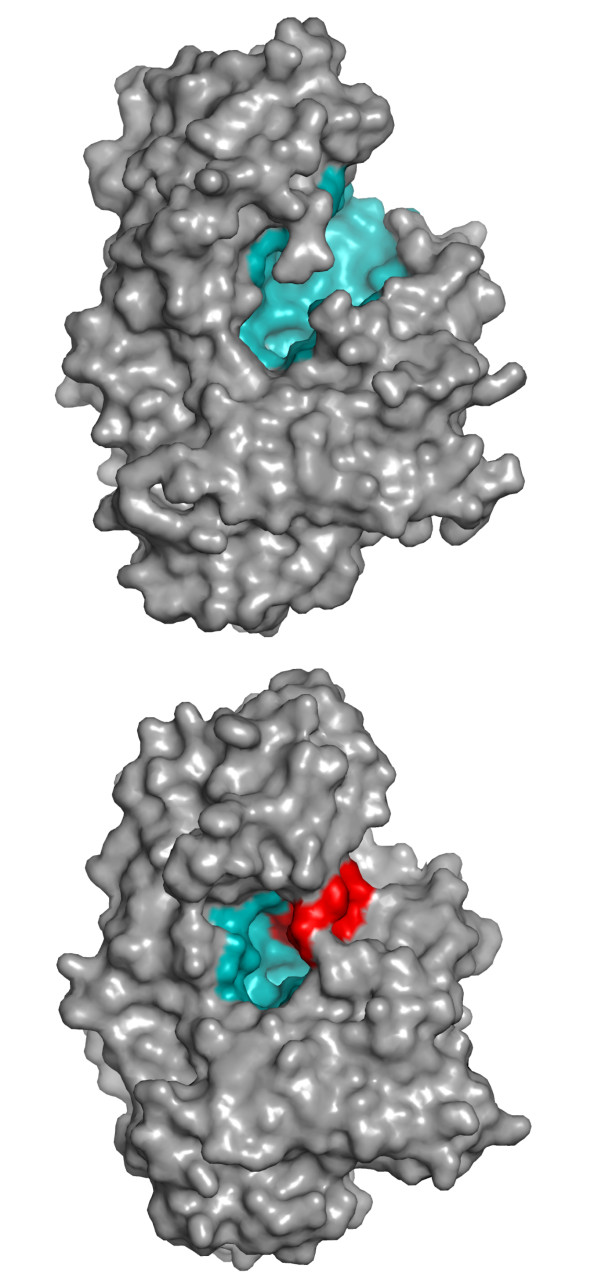
**The basis for the orthogonal substrate specificities of PRCP and DPP7**. PRCP (top) and DPP7 (below), showing the substrate-binding groove of PRCP (blue), and the substrate-binding site of DPP7 (blue) that is restricted by the hairpin insert (red) [1]. The carboxy-terminal side of the catalytic site (see text for discussion) is to the left in this illustration.

The orthogonality between the PRCP carboxypeptidase and the DPP7 aminopeptidase activities is at least partly explained by a single peptide insertion sequence in DPP7. The resulting disulfide-stabilized, short hairpin structure sits in the substrate-binding groove of DPP7, just beyond the proline S1 binding site, blocking the binding of substrates beyond the S2 binding pocket (Figure 2). In other words, the restricted binding groove can only accommodate an X-Pro sequence on the amino-terminal side of the hydrolytically sensitive peptide bond. The result is an enzyme that is sterically restricted to a dipeptidyl peptidase activity with specificity for proline at the S1 binding pocket. The hairpin also introduces an Asp residue, which may be important in binding the free amino-terminal amino group of the substrate in the S2 binding site, in a manner reminiscent of other DPPs, such as DPP4.

For PRCP, the absence of the hairpin insert opens up the substrate-binding groove for longer amino-terminal peptides (Figure 2), thereby loosening substrate specificity towards the carboxyl terminus. It does not, however, completely explain the shift to the specific carboxypeptidase activity of PRCP. The binding groove on the carboxy-terminal side of the catalytic site appears to be open, as in the DPP7 structure (Figure 2), but carboxy-terminal specificity is restricted to a single amino acid beyond the S1 proline, creating an exo-carboxypeptidase. It is not immediately obvious why PRCP does not have a more pronounced endopeptidase activity. This suggests that there are probably other substitutions in the PRCP binding groove that restrict the carboxy-terminal peptide length. Whatever those substitutions are, they are much less dramatic than the amino-terminal blockade that creates the DPP7 aminopeptidase specificity.

The orthogonal substrate specificity noted for PRCP and DPP7, that is, carboxypeptidase versus aminopeptidase activities, suggests a functional discord between the pair; this is an illusion. The proline binding at the S1 site is essentially identical for both enzymes and both reactions are catalytically superimposable, with hydrolysis always occurring at the proline carbonyl. The specificity shift appears to be driven by binding forces and restrictions that dictate which end of a peptide each enzyme can accommodate; the enzyme chemistry for both is the same.

As noted earlier, the properties of PRCP might make it a potential candidate for a drug target in the treatment of hypertension and inflammation. The treatment of hypertension is generally well served by a large variety of commercial drugs, many now generic, with different mechanisms of action. The bar for the entry of new drugs into that space is high; a focus on the anti-inflammatory and cardioprotective potentials of PCRCP may be a better approach. Clear evidence of differentiation from existing therapies and significant add-on benefit will be critical to capture the developmental interest of major pharmaceutical companies. The therapeutic potential of DPP7 (also known as DPP2 and QPP) is much more tenuous. Because DPP7 inhibition induces a novel apoptotic pathway in quiescent lymphocytes, the potential for drugs targeting the enzyme in some cancers is interesting, albeit controversial [7]. In addition, tissue-restricted deletion of DPP7 leads to metabolic defects, suggesting that it may play a novel role in glucose regulation [8]. Studies with potent, specific DPP7 inhibitors have given mixed and, at times, conflicting results [9,10]. Regardless of the prospects of PRCP and DPP7 as viable drug targets, the new insights into serine proteases gleaned from the study of the S28 family will undoubtedly refine our molecular understanding of this classic mechanism of covalent catalysis.
